# Shifting to online and telephone bereavement support provision during the COVID-19 pandemic: A mixed methods study of bereavement service provider perspectives and lessons learnt for current practice

**DOI:** 10.1177/02692163251383324

**Published:** 2025-11-24

**Authors:** Lucy E. Selman, Jenny Birchall, Eileen J. Sutton, Tracey Stone, Renata Medeiros Mirra, Emma Gilbert, Mirella Longo, Kathy Seddon, Anne M. Finucane, Alison Penny, Anthony Byrne, Emily Harrop

**Affiliations:** 1 Palliative and End of Life Care Research Group, Population Health Sciences, Bristol Medical School, University of Bristol, UK; 2 Cardiff School of Dentistry, Cardiff University, UK; 3 Marie Curie Research Centre, Division of Population Medicine, School of Medicine, Cardiff University, UK; 4 Wales Cancer Research Centre, Cardiff, UK; 5 Marie Curie Hospice Edinburgh, Edinburgh, UK; 6 Clinical Psychology, School of Health in Social Science, University of Edinburgh, Edinburgh, UK; 7 National Bereavement Alliance, London, UK

**Keywords:** grief, pandemics, bereavement, coronavirus infections, bereavement services, palliative care, telemedicine, digital health

## Abstract

**Background:**

Provision of remote (online/telephone) bereavement support accelerated during the COVID-19 pandemic. However, the extent and impact of this change and lessons learnt are unknown.

**Aim:**

To determine the extent to which UK voluntary and community sector bereavement services moved to remote support provision during the pandemic, explore providers’ perspectives on this shift, and consider implications.

**Design:**

Mixed methods explanatory sequential study, conducted spring 2021: (1) Online survey of UK bereavement services; (2) Qualitative interviews with staff and volunteers.

**Setting/participants:**

147 services participated in the survey; 44.5% hospice/palliative care services; 15.1% national charities/organisations; 11.6% local charities/ organisations. 24 interviews were conducted across 14 services.

**Results:**

Pre-pandemic, remote support was offered by <10% of bereavement organisations. By spring 2021, there had been increases in online: peer group meetings (3.4% pre-pandemic to 33% during, OR 13.8), facilitated group meetings (4.1%–56%, OR 30.48), 1:1 support (8.8%–83%, OR 50.3), and specialist intervention (3.4%–36%, OR 16.01). Telephone bereavement support was also more widely available. The appropriateness and acceptability of these changes differed by client group. Adaptations presented organisational/logistical challenges, and difficulties for support providers working from home. Smaller organisations with fewer resources found these harder to accommodate. Hybrid working and new technologies were reported to increase service efficiency and cost-effectiveness.

**Conclusions:**

Remote delivery of bereavement support increased support capacity and can potentially reduce inequities in access. However, it needs to be carefully tailored, and is not appropriate for everyone. Staff and volunteers providing remote services require training and support.

**What is already known about the topic?**

- The COVID-19 pandemic increased the demand on bereavement support services while the provision of in-person support was severely restricted, necessitating a shift to remote (online and telephone) provision.
- The extent to which bereavement services changed their services to remote provision, and the impact of this on organisations, staff and volunteers, is not well understood.

**What this paper adds?**

- There was a dramatic shift to remote support provision, particularly online and telephone 1:1 and group support, with innovations including online coffee mornings, storytelling and mindfulness.
- Online support was preferred by some (particularly men and younger people) and increased reach in rural communities, however it was less appropriate for older people, very young children, people with communication difficulties or with very high/complex needs.
- Adaptations presented organisational and logistical challenges, particularly for smaller organisations, and challenges for staff and volunteers providing bereavement support from their own homes, but were reported to increase access, efficiency and cost-effectiveness.
- The adaptations also had positive organisational outcomes including greater inter-organisational connectivity and reach, support opportunities for staff and an impetus to modernise other areas of practice.

**Implications for practice, theory or policy**

- Online bereavement support might hold potential in reducing inequities in access to bereavement support, however it needs to be carefully designed for different client groups and is not appropriate for everyone; further research is needed to assess its acceptability and feasibility in diverse population groups.
- Staff and volunteers providing remote services require training and support, including in relation to the psychological impact of home working; organisations need to prioritise opportunities for team members to connect and sustain each other.
- Study findings should inform future development of remote models of bereavement support, which were emerging before the COVID-19 pandemic, but have increased rapidly since.

## Background

During the COVID-19 pandemic, excess mortality increased demand for voluntary and community sector bereavement services, which play a crucial role in bereavement support but were already known to be stretched.^1–3^ The sudden and unexpected nature of many deaths, lack of access to and physical contact before a death, funeral restrictions and isolation measures caused high levels of distress, increasing bereavement support needs and levels of prolonged grief disorder.^4–7^ Globally, many countries have seen sustained excess all-cause mortality post-pandemic.^8–11^ Even before the pandemic, bereavement support was highly variable and frameworks for the commissioning and delivery of bereavement services often lacking or inconsistently implemented, in the UK, other European countries and Australia.^2,12–14^ Barriers to accessing bereavement support include a lack of specialist support, long waiting lists, restrictive eligibility criteria, unclear referral pathways and geographical variability.^13,15–19^ Bereavement support has been highlighted as an international public health priority,^20^ and is a core component of palliative care.^21^

Bereavement services have historically offered provision encompassing structured activities, and direct (personal contact) and indirect (information provision) support.^22–25^ In 2020, however, services had to rapidly adapt to meet changing demand and modify their support to comply with widespread physical distancing measures. In particular, while provision of remote support (online and/or via telephone) was increasing pre-pandemic, this accelerated rapidly during the pandemic,^13,26–28^ with lasting effects. The UK Commission on Bereavement, for example, reporting in 2022, described the move to providing online and virtual bereavement support as a ‘paradigm shift’ (p.127), reporting that services had retained a ‘blended approach’ to support provision.^29^ There is growing evidence that online provision, which includes video-conferencing, websites, apps and other virtual resources, can widen access to safe, effective bereavement support and improve bereavement outcomes.^28,30–33^ However, access to online services differs across populations, with some groups experiencing digital exclusion.^34^ During the pandemic, groups with lower digital literacy or without necessary resources to access digital devices faced intensified barriers to accessing support and information,^35^ which may intersect with and compound other inequities.^17–19^ It is therefore crucial that we better understand perspectives on the shift to remote provision instigated by the pandemic and how it influenced access to support.^15,18,36,37^

Evidence from healthcare and mental health services has highlighted how, at a time when rapid and radical changes to services were being implemented, service providers faced multiple challenges related to practitioner training,^38^ the adverse impacts of COVID-19 on staff,^39,40^ and providing and using new forms of remote support.^41–43^ Little is known, however, about the experiences of bereavement service providers during this time or how lessons learnt during the pandemic can inform future practice. Given the continued expansion of remote support provision in bereavement and palliative care post-pandemic,^29,44^ this evidence is vital to inform the design and delivery of remote bereavement support models.

We aimed to determine the extent to which bereavement services moved to remote support provision during the COVID-19 pandemic and explore providers’ perspectives on this shift.

## Methods

A pragmatic, explanatory sequential mixed methods study^45^ comprising:

- An online cross-sectional open survey of voluntary and community sector bereavement services in the UK, disseminated via UK-based national organisations, networks and social media (March–May 2021).
- Qualitative semi-structured telephone interviews with staff/volunteers at selected bereavement services (June-December 2021) which aimed to expand on the survey findings.

Here we present findings related to the provision of remote (online and telephone) bereavement support, using the Checklist for Reporting Results of Internet E-Surveys^46^ in reporting. This work is part of a larger research study which also examined experiences of bereavement during the pandemic in the UK.^4,7,15,37,47^

***Setting and population:*** Voluntary and community sector bereavement services in the UK. In the UK, voluntary and community sector services provide the bulk of bereavement support, free to bereaved people. Services are highly diverse, ranging from small local organisations to large national ones; from those working with all bereaved people to those targeted to a particular group such as parents bereaved of a child or widows. Services provided may be universal, targeted or specialist, reflecting the tiered approach to bereavement^48,49^; can involve practical and/or emotional support; and can be provided in groups or 1:1.^50^ There are disparities in the availability of bereavement services across the country.^19^

### Sampling

*Survey:* Convenience sample of voluntary and community sector bereavement services.

*Qualitative interviews:* We purposively sampled 14 bereavement support organisations from the 147 organisations who completed the online survey. Sampling captured diverse organisations and experiences, considering: organisation size; geographical area; type of support provided; support for specific groups (e.g. minoritised ethnic communities, children and young people); reported challenges and innovations during the pandemic. In addition, we included two UK social media communities providing support to people bereaved during the pandemic, as these were an important source of support which was not captured in the survey.

### Recruitment

*Survey:* A link to a JISC^51^ survey was disseminated to voluntary and community sector bereavement services, via emails from the research group and national bereavement organisations and associations, national stakeholder webinars and social media, and posted to the study website. We asked one representative from each organisation to participate, consulting with colleagues as needed.

*Qualitative interviews:* Sampled participants (one at each selected organisation) were sent an invitation, information sheet and consent form. After the initial interview, the team decided whether or not to interview additional staff/volunteers from the organisation, considering the data collected and the size and nature of the organisation. These interviews aimed to capture additional perspectives, for example, manager/team lead in addition to bereavement counsellor. All participants gave written consent.

### Data collection

*Survey:* The survey (Supplemental File 1) comprised non-randomised open and closed questions exploring the impact of the pandemic on bereavement services and their response. Survey items were based on the literature and initial scoping of the pandemic’s impact,^13,26^ with input (including testing) from an expert advisory group of researchers, clinicians, bereavement support practitioners and people with experience of bereavement. Two participants completed the survey twice; their first and second responses were merged. Two services provided two responses; the second response from each was excluded.

*Qualitative interviews:* Telephone interviews were conducted using a semi-structured topic guide (Supplemental File 2; adapted for online services), developed as above. Interviews were conducted by EJS (*n* = 21), EG (*n* = 2) and LES (*n* = 1), experienced qualitative researchers. Fieldnotes were taken to inform sampling, data collection and analysis.

### Analysis

*Survey:* All quantitative data are categorical. Graphical summaries, including pie charts, bar charts and stacked bar charts, were used to describe all variables. Analysis was performed by RMM using R (version 4.1.1, R Core Team, 2021), implemented in R-Studio (www.r-studio.com). Free-text data were analysed using thematic analysis^52,53^ in NVivo12^54^ by TS, discussed with LES and EJS and refined by all co-authors.

*Qualitative interviews:* Interviews were transcribed verbatim and checked for accuracy prior to thematic analysis^52,53^ in NVivo12.^54^ Analysis used a combination of deductive and inductive coding strategies and was conducted concurrently with data collection, allowing insights from earlier interviews to inform those conducted subsequently. EJS, LES and TS read and independently coded a sub-set of interview transcripts and developed a coding framework which EJS applied to the dataset. EJS and LES met regularly to discuss the development and revision of key themes and sub-themes,^55^ drawing out differences, similarities and patterns in the data.

Quantitative and qualitative findings were triangulated and integrated into a narrative (JB, LES), with the latter used to explain and add richness to quantitative findings.^45^ All quotations are anonymised, with pseudonyms used in data extracts.

### Ethical approval

Ethical approval for the study was granted by the University of Bristol, Faculty of Health Sciences (Ref: 114304 20/12/2020).

## Results

### Participants

**Figure 1.**
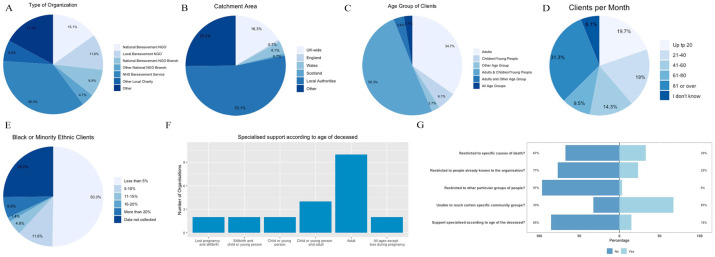
Organisation characteristics from survey (n = 147).

*Survey:* Participants represented 147 bereavement services from across UK regions (Figure 1). As this was an open survey the response rate is not known. 44.5% were hospice or palliative care services (including services part-funded by the NHS); 15.1% national bereavement charities or non-governmental organisations (NGOs); 11.6% local bereavement charities/NGOs; 8.9% branch of a national bereavement charity/NGO; 4.1% branch of other national charity/NGOs; 6.8% other local charities/NGOs; 8.9% other (e.g. council-commissioned service, local collaborative partnership, or community interest company). 68% provided support following all causes of death; 32% were focused on specific causes of death such as terminal illness.

*Qualitative interviews:* Twenty-four interviews with staff and volunteers from 14 organisations were conducted (Table 1). Interviews lasted 25–77 min (mean 46 min).

**Table 1.** Details of organisations and interview participants.

Organisation ID	Size	Type of service	Group(s) supported	Bereavement services provided (pre-Covid unless stated)	Geographical area	Interviewee role
Org A	Regional	Hospice	Bereavement support for children and young people (and those caring for them) and adults following all causes of death	Information on grief and sign-posting to other services; group meetings of peers; group meetings facilitated by someone with training; one-to-one support e.g. counselling Other services: Pre-death support Immediate post-death support Remembrance services Drop-in support	South East England	A1: Manager A2: Director
Org B	Small	Bereavement charity mainly supporting minoritised group (Muslim community)	Bereavement support for children and young people (and those caring for them) and adults following all causes of death	Information on grief and sign-posting to other services; group meetings facilitated by someone with training; one-to-one support e.g. counselling Other services: Pre-death support Drop-in support	UK-wide	B1: Director B2: Volunteer
Org C	Branch of National Organisation	Bereavement charity	Bereavement support for children and young people (and those caring for them) and adults following all causes of death	Information on grief and sign-posting to other services; group meetings of peers; group meetings facilitated by someone with training; one-to-one support e.g. counselling Other services: Pre-death support	West Midlands	C1: Regional Manager C2: Volunteer
Org D	Small	Bereavement NGO supporting minoritised group (African Caribbean community)	Bereaved adults following all causes of death	Information on grief and sign-posting to other services; group meetings facilitated by someone with training; one-to-one support e.g. counselling	UK-wide	D1: Director
Org E	Regional	Hospice/palliative care service part-funded by NHS, Regional Bereavement Network	Bereavement support for children and young people (and those caring for them) and adults following death from a life-limiting illness	Information on grief and sign-posting to other services; group meetings of peers; group meetings facilitated by someone with training; one-to-one support e.g. counselling; specialist intervention involving mental health services/specialist counselling Other services: Pre-death support Immediate post-death support Condolence letters Home visits Remembrance services Support with funerals Walking group Gardening group	Scotland	E1: Team Lead E2: Volunteer
Org F	Small, Regional	Bereavement charity, Regional Bereavement Network	Bereavement support for children and young people (and those caring for them) following all causes of death	Information on grief and sign-posting to other services; group meetings of peers; group meetings facilitated by someone with training Other services: Pre-death support Immediate post-death support Home visits Drop-in support Online community Activity days	Scotland	F1: Coordinator
Org J	Regional	Charity	Bereavement support for adults following death from a life-limiting illness	Group meetings facilitated by someone with training; one-to-one support e.g. counselling Other services: Individual and group therapy for people facing death and those close to them	South West England	J1: Clinical Lead J2: Senior Therapist
Org K	Regional	Hospice/palliative care service part-funded by NHS	Bereavement support for children and young people (and those caring for them) and adults following all causes of death	Information on grief and sign-posting to other services; group meetings of peers; group meetings facilitated by someone with training; one-to-one support e.g. counselling Other services: Pre-death support Immediate post-death support Home visits Remembrance services	North East England	K1: Senior Practitioner K2: Volunteer
Org P	Branch of National Organisation	Palliative care charity	Bereavement support for adults known to the palliative care service following death from a life-limiting illness	Information on grief and sign-posting to other services; group meetings of peers; group meetings facilitated by someone with training; one-to-one support e.g. counselling; specialist intervention involving mental health services/specialist counselling Other services: Pre-death support Immediate post-death support Condolence letters Home visits Remembrance services	South East England	P2: Specialist Counsellor
Org N	Small, Regional	Hospice	Bereavement support for children and young people (and those caring for them) and adults following all causes of death	Information on grief and sign-posting to other services; group meetings of peers; group meetings facilitated by someone with training; one-to-one support e.g. counselling; specialist intervention involving mental health services/specialist counselling Other services: Pre-death support Immediate post-death support Home visits Remembrance services Drop-in support Bereavement education for professionals Bereavement sessions in schools and community groups	Wales	N1: Team Lead N2: Social Worker
Org M	Branch of National Organisation	Bereavement charity	Bereavement support for children and young people (and those caring for them) and adults following all causes of death	Information on grief and sign-posting to other services; one-to-one support e.g. counselling	Northern Ireland	M2: Regional Manager M3: Volunteer
Org Q	National Organisation	Palliative care charity	Telephone bereavement support for adults following death from a life-limiting illness	Information on grief and sign-posting to other services; one-to-one support e.g. counselling Other services: Online community	UK-wide	Q1: Coordinator Q2: Volunteer
Org H	Online Organisation	COVID-specific social media group (non-profit)	Online bereavement support for adults following a death from COVID-19, with counsellors moderating the group	Information on grief and sign-posting to other services; online group meetings of peers; online group meetings facilitated by someone with training Other services: Online community support Training for professionals	UK-wide	H1: Founder
Org L	Online Organisation	COVID-specific social media group (non-profit)	Online peer-to-peer bereavement support for adults following a death from COVID-19	Information on grief and sign-posting to other services; group meetings of peers Other services: Online community support Memorial services	UK-wide; Wales	L1: Founder L2: Regional Administrator

NGO: non-governmental organisation; NHS: National Health Service (UK).

### Findings

#### The rapid shift to remote provision

Before the pandemic, the most common services provided were those involving face-to-face support, while the least common services were online support or any telephone support besides information and signposting services (Table 2). By the time of the survey (spring 2021), face-to-face support provision of all types had decreased rapidly while online and telephone services had increased (Table 2).

**Table 2.** Comparison of services provided before and during the pandemic.

Services provided	Pre-pandemic % (*n*)	Post-pandemic % (*n*)	OR
In-person information on grief and sign-posting to other services	83.0 (122)	21 (31)	0.05
In-person group meetings of peers^a^	50.3 (74)	4.1 (6)	0.04
In-person group meetings facilitated by someone with training	77.6 (114)	11 (16)	0.04
In-person 1:1 support	87.1 (128)	27 (40)	0.06
In-person specialist intervention^b^	44.2 (65)	16 (24)	0.25
Online information on grief and sign-posting to other services	31.0 (46)	80 (117)	8.56
Online 1:1 support	8.8 (13)	83 (122)	50.3
Online group meetings facilitated by someone with training	4.1 (6)	56 (83)	30.48
Online peer group meetings^a^	3.4 (5)	33 (48)	13.77
Online specialist intervention^b^	3.4 (5)	36 (53)	16.01
Telephone information on grief and sign-posting to other services	70.5 (104)	86 (127)	2.63
Telephone group meetings of peers^a^	4.1 (6)	9.5 (14)	2.47
Telephone group meetings facilitated by someone with training	2.0 (3)	9.5 (14)	5.05
Telephone 1:1 support	41 (60)	85 (125)	8.24
Telephone specialist intervention^b^	14 (21)	40 (59)	4.02

a People with similar experiences but no one is trained.

b Involving mental health services, psychological support services or specialist counselling psychotherapy.

Interviewees reported how staff and volunteers worked together to rapidly instigate these changes to support provision at the start of the pandemic, shifting support away from face-to-face delivery to online and via telephone:

When the pandemic hit the first thing we did, we just transferred everything online. It was so hard. I know there’s been lots of training now . . . about what to do and what not to do. I think we applied a lot of common sense and what we were saying to the clients that we were offering them . . . Zoom, WhatsApp or telephone support because we weren’t at that point in time able to offer any face-to-face stuff. (E1: Patient & Family Support Lead, Hospice)

Some services started delivering online group support so that they could cope with an influx of new clients needing support at an early stage:

. . . so we did calls, six support calls one-to-one. And then we did six groups, like what we would normally do groups, but it was a bit different because the losses were quite recent, too recent to do what we would usually do. Because when we did our group support before COVID, we wouldn’t take anyone who’d had a loss before sort of 3-6 months. (D1: Director, Small Org supporting specific ethnic community)

Survey participants also described other new initiatives reaching out to service users in innovative ways that complied with lockdown and social distancing measures, including online coffee mornings and mindfulness sessions, virtual storytelling sessions for children and social media activities/groups:

Our tele-friending project has been invaluable to those bereaved over this time, with so many changes to social contact, funerals etc, this is a weekly check in call to help clients set a few targets for the coming week, gently challenge some of their thinking and signpost them as required. (Survey ID130, Branch of national bereavement charity/NGO)

We developed pre and post bereavement packs for children, which were filled with craft resources, books and games, were dropped off at family’s homes. Follow up video sessions were then arranged with the practitioner and family working through the materials in the packs together. (Survey ID90, Director of Care Service)

#### Accessibility and appropriateness of remote support

The shift to remote provision changed the accessibility of support and had differing degrees of appropriateness and acceptability across population groups. Online and telephone support was reported to be well-accepted and even preferred by some clients, including younger people and men:

. . . if you’re in not a very good place and you’re grieving and feeling a bit overwhelmed then not having somebody watch you as you’re falling apart is easier for some people to deal with as well. (B2: Befriender, Small Org supporting specific ethnic community)

Most of the teenagers that we’ve had referrals in for opt for online counselling, so they don’t mind doing Zoom, Teams. (A1: Regional Hospice, Manager)

Organisations noted that diversifying their services meant that they were now able to support more people in their local community, particularly in rural areas where access to face-to-face services was previously difficult:

The anticipated increased need for bereavement support as a result of COVID has encouraged us to further extend our offering of services wider to the people of [region]. (Survey ID8: Local other charity/NGO)

Support providers working in rural areas reported that they were able to schedule more appointments as travel time was reduced or eliminated for both clients and support providers. One organisation also drew attention to positive aspects for some Muslim women grieving the loss of their husbands:

. . . because of the circumstances that we’re in and for some women, it might be the only way that they can have support. So, for example, if they’ve got a young baby. Muslim women tend to go through something called the Iddah period after they lose their husband where, for four months and 10 days, it’s a period of reflection where they tend to stay at home, and for them to access this support is now a possibility, so this idea that it is possible to get support remotely. (B1: CEO, Small Org supporting specific ethnic community)

However, changes to services were described as problematic for some older people and those with communication difficulties, leading to decreased engagement:

I think, for some people if you’re struggling a bit with your hearing or your sight, you know peering at a screen and trying to make sense of it. It can be really tough and I think you know, just that face-to-face connection again – people have really missed that. (K1: Senior Practitioner, Hospice)

Digital exclusion, and the difficulty of alleviating it in the context of pandemic-related restrictions, was also a problem, especially for those working in deprived areas or with older people:

It’s an inequitable service . . . there’s lots of people that couldn’t [use technology] . . . you couldn’t even have bereaved relatives being visited by grandchildren, for example, to set the tech up for them. . . for some of the other virtual groups we’ve done at the hospice, we’ve had somebody that’s gone around to try and set things up initially and we had a couple of iPads around, but the difference between fantasy and reality is huge sometimes. (N1: Family Support Team Lead, Hospice)

It’s more to do with accessibility for people who are digitally poor – who do not have access to tech or confidence or training on how to use/access the support service over Zoom for example.’ (ID32: Branch of national charity (Wales))

Interviewees described how working with clients online, or via the telephone, impacted upon the therapeutic encounter, explaining how work with some groups, such as very young children, was adversely affected:

The people that we think have missed out the most are the young, the children . . . that we haven’t really been able to engage with over this time . . . apart from . . . sending out some resources, but that’s tricky even if a child has lost a parent and the other parent is grieving as well, it’s hard for her to sit down and do the necessary, the therapeutic activity work with their children. (M3: Volunteer, Branch of Nat Org)

The children’s group met for a while via Zoom but it was not successful - the children were not able to engage with new people they had never met before via a Zoom call. (Survey ID101: hospice/palliative care team bereavement services part-NHS funded)

Nevertheless, participants also described innovative ways of working online with young children using ‘show and tell’ techniques, and making use of children’s confidence with technology.

Working with clients with high emotional support needs, or those who were referred from mental health services was also viewed as especially challenging:

. . . they [staff/volunteers] have to use extra listening skills and reading between the lines because they can’t see the person, they can’t see what’s going on with them. . . If they’ve got suicidal ideation, you can’t see any of that on the telephone. (A1: Head of Wellbeing, Hospice).

To ensure that remote services were safe and worked well for service users, organisations needed to think carefully about how to set up and run them, including supporting clients to establish a private space free from interruption and distraction:

We are still in the process of developing an online group offer which has required some careful planning in terms of safeguarding and forming group relationships online. (Survey ID18, national charity branch/NGO)

#### Challenges of providing remote support from home

Alongside the shift to remote support provision, most bereavement services’ staff and volunteers started providing support from their own homes. Interviewees reported a number of organisational challenges related to these changes, including putting IT/telephone systems in place, ensuring good connectivity, funding extra equipment and IT support and adapting policies and procedures, particularly for safeguarding:

We had to get them all telephones and we made sure that they were password protected and how the processes of making sure that when they’re finished with a client all the details are taken off in line with data protection, so there was quite a lot of. . . work but you just work through it. (E1: Patient & Family Support Lead, Hospice)

Once equipment was procured and new working practices established, organisations experienced other challenges around supporting staff and volunteers, including delivering training in providing support when usual social cues and facial expression are not available, the potential emotional impact and interacting as a team. To cope with the demands of the pandemic and providing therapeutic support from home, additional clinical supervision, staff and volunteer support and self-care were seen as essential:

An unexpected consequence of working from home is the emotional impact of the work we do has entered our homes. When working from the hospice, the impact can be left there, or shed on the drive home. That isn’t possible and staff have noticed that they need to manage their session time in a different way, maybe going for a walk or regular breaks. (Survey ID6, hospice/palliative care service part NHS-funded)

Participants reported the benefits of staff sharing their experiences:

If you’ve had a tough client session on the phone, you put the phone down and then you’re sitting on your own staring at the fire and it’s triggered your own thoughts, that’s really tough . . . being able to say look, you know give us a ring, have an offload afterwards, how are you doing? . . . we always say to clients there’s a massive strength in showing your vulnerability… But of course that also applies to us. (E1: Senior Practitioner Family Support, Hospice)

Impacts across organisations differed. Some staff and volunteers enjoyed working from home, whereas others had stepped back as they felt unable to continue the work under the changed circumstances of the pandemic, necessitating further recruitment:

Some bereavement volunteers stepped back . . . [they] did not want to support over the phone or by Zoom, so we’re now looking at refresher training for volunteers who may want to come back once we’re introducing in person support again. (C1: Hub Manager, Branch National Org)

It’s been okay. I’m quite lucky in the sense that at home . . . I do have the space to be able to close the door and make phone calls and take phone calls and I don’t have really young children, so my times are quite flexible . . . I know other people have struggled especially when they’ve had COVID in the family and have lost loved ones themselves. (B2: Befriender, Small Org supporting specific ethnic community)

Staff were simultaneously facing challenges arising from providing bereavement support during the pandemic, and negotiating the impacts of the pandemic on their own lives:

I have seen greater levels of anxiety and depression within the team. This is, I believe, influenced by a number of factors including the nature of the work, but also, lockdown, caring for children at home, restrictions on your life and activities, having your workspace in your home, being flexible in your approach, learning new skills and ways of working. A big ask! (Survey ID6: hospice/palliative care bereavement service, part NHS-funded)

Smaller organisations with fewer resources found these adaptations and challenges particularly difficult to accommodate:

The IT department had to change really, put all this in place, and we’ve only got a small IT department. Plus, in the children’s service everybody is young and quite good with IT stuff; in our adult counselling service we’ve got five paid members of staff, three of whom are really scared of IT because they’re older and . . . probably half of [our volunteers] are quite scared of IT as well, so it was actually reassuring and training and being there for staff as well to support them through the changes as well. (A1: Head of Wellbeing, Hospice)

National/larger organisations were reported to have more resources available to them to facilitate service changes. These included more stable funding, staff resources, IT infrastructure and access to COVID-safe spaces. Small organisations felt the loss of funding streams more acutely and were more dependent upon volunteers, so felt a greater impact in the event of staff/volunteer sickness or departure. Some services were already providing support by telephone and hence were able to transition more easily away from in-person support.

Interviewees were asked if any additional support would have been helpful to them throughout the pandemic. Smaller organisations highlighted the lack of technological resources and support which they encountered when moving away from in-person provision:

Um [laugh] someone sitting next to me helping me with the tech [laugh] but that wasn’t ever gonna happen. [laugh] Well that’s not true, that’s not true. I had a son who was quite helpful at that point. (J2: Senior Therapist, Regional Org, Counselling)

#### Positive impact and lessons learnt

There was universal agreement that the modernisation of services, although challenging, had positive implications for future provision. Respondents reported that the pandemic had given them an unexpected opportunity to review their working practices and reassess how best to support their clients:

It has given us a chance to evaluate the service, looking at what works and what doesn’t and has given us an opportunity to explore other methods of delivering support. (Survey ID12, hospice bereavement service)

Respondents described valuable learning about how to work effectively online, both as an organisation and in terms of client support, which improved their service:

The move to online working (support/counselling/groups) has been a challenge in terms of learning, systems, protocols, processes, support for the team. However, we have learnt to work this way and it has been positive in providing a service at a difficult time. We’ve also learnt that online work has a value and purpose and that face-to-face is not the only way! (Survey ID6, hospice bereavement service)

[We have] greater use of social media - e.g. WhatsApp with young people, using Zoom and allowing for cameras to be off and the use of pseudonyms to anonymise those who want support but do not want to be identified - especially young people and those with a background of complicated social circumstances. (Survey ID119, National bereavement charity NGO)

Respondents reported benefits in terms of efficiency and cost saving:

We have had to accelerate IT changes that we planned - moving to online referrals, using Microsoft Teams, Zoom, webinars for training. Many of these have cost saving implications for the future and have streamlined our processes. (Survey ID56, Local bereavement charity/NGO)

Many survey respondents reported that they would continue to provide a more diverse range of services, while recognising that for some client groups, in-person services are still the preferred option:

As our online work has been so well received and delivered, we now have the opportunity to continue to work online as well as face -to-face, offering our families a greater choice as to how and when they receive their bereavement support.’ (Survey ID58, Children’s hospice)

Similarly, all the organisations represented in the qualitative interviews intended to use a blended approach in the future to provide clients with greater choice, recognising the improvements in accessibility:

Going forward, [National Organisation M] are offering now a range services for those. . . groups that prefer telephone, Zoom or for those people who want to see another human in a room, that is also available. So there’s more choice now in the way we deliver our service. (M2: Hub Manager, Branch of National Org)

Survey respondents discussed how the use of technology provided support and solidarity for staff and volunteers:

We kept in touch remotely, with video and telephone calls between supervisors, and between supervisors and volunteers, and between volunteers themselves (including WhatsApp groups for sharing the funny stuff as well as the tricky stuff). (Survey ID121, Local bereavement charity/NGO)

The increase in online working and communication allowed organisations to develop greater connectedness with other local and national services:

It has been positive to work with other local charities and organisations. Connections are not only local, but Twitter/Facebook have enabled wider contact, sharing of knowledge, reduction in barriers. (Survey ID80, Community-led peer support)

## Discussion

### Main findings

This study offers new and unique insights into how voluntary and community sector bereavement service providers in the UK rapidly adapted their services to provide remote support during the pandemic, and the lessons learnt as a result of these adaptations. In this regard, the pandemic was an opportunity to innovate and a testing ground for new approaches to bereavement support provision that had commenced prior to the pandemic but which increased dramatically during it, with lasting effects on the sector.^29^ Given that bereavement and palliative care services continue to widely utilise remote methods of support, often driven by the need to tackle inequities in access, these findings are relevant to and should inform current practice.^56–59^

The shift to remote provision and reduction of face-to-face support reported by service providers changed the accessibility of support, with differing degrees of appropriateness and acceptability across different population groups. Remote support was reported to be well accepted and even preferred by some clients, such as young people, but changes to services were problematic for some older people, very young clients and people with communication difficulties, leading to decreased engagement. These findings align with those of a recent systematic review, which describes the varying degrees of acceptability of online bereavement support in diverse contexts and settings internationally.^28^ Interviewees reported logistical challenges related to the changes in service provision, as well as challenges around supporting staff and volunteers, some of whom found it difficult to provide bereavement support while negotiating the impacts of the pandemic on their own lives. Smaller organisations with fewer resources, many of which were hospices, found these adaptations and challenges particularly difficult to accommodate, while national bereavement services and larger organisations generally had more resources available to them to facilitate changes to provision. However, there was agreement that the pandemic-forced modernisation of bereavement services, while challenging, had positive implications for future service provision. Respondents reported that they would continue to provide a more diverse range of services in future, while recognising that for some client groups, face-to-face services are still preferred.

### What this study adds

Our findings resonate with previous studies of remote care provision before and during the pandemic. Participants described the cost- and time-savings of remote provision and working from home,^60^ and the flexibility and ease of access which this affords service users^38,42,61^ who adapt to using remote services.^13,26–28^ However, there are also drawbacks to remote support. Corroborating evidence in mental health support,^41^ remote provision was reported to affect the therapeutic relationship and practitioners’ ability to identify and respond to non-verbal cues, which was a particular concern in clients with complex or mental health needs. There were also similar challenges related to safeguarding, safety and privacy.^42^ Online methods disadvantage people less confident or proficient in technology, including older people,^62^ people with sensory or cognitive impairment, and those who are digitally excluded^42^ due to socio-economic deprivation. Over-reliance on remote provision could hence potentially compound known inequities in bereavement support. Clients may need support to access and use technology, and flexibility regarding types of technology or platform, as well as the continued option of face-to-face support.^42,63^

We found that given the speed at which social distancing restrictions were introduced, it was difficult to fully train and prepare practitioners, adding to wider evidence of the pressure that support services were under.^38,41^ Supporting previous findings,^13,26,27,39,40^ we found that experiences of providing and using new forms of remote support could have a considerable impact on the mental health and wellbeing of staff and volunteers, leading some volunteers to step away from the service. This had a major impact on smaller services, including many hospices, which rely heavily on volunteers.^64^ Despite the challenges of changing to remote provision, respondents reported benefits including enhanced working practices and increased collaboration.

### Limitations

Convenience sampling might have resulted in less burdened or more engaged services completing the survey. It is not known precisely how many voluntary and community sector bereavement services there are in the UK; a 2020 analysis of services registered on a national directory identified 822 entries,^13^ however this is likely to include services outside the sector and services no longer operating.

### Implications for research

Research is needed to: (a) further understand the acceptability and outcomes of online support in different client groups, particularly perspectives amongst under-served groups and the unintended consequences and risks to equity of delivering support exclusively online; (b) establish good practice in the provision of remote bereavement support, including in the context of palliative care, which is sensitive to the needs of different client groups and cognisant of the impact and training needs of service providers; and (c) map and categorise bereavement service delivery models since the pandemic and compare their impact, using a realist lens to understand the mix of models currently being offered and the suitability of these in specific populations and contexts.

### Conclusions and implications for policy and practice

Study findings demonstrate the dramatic shift to remote bereavement support during the pandemic, and highlight service provider perspectives on and experiences of this shift and its impact. Online support provision has both benefits and drawbacks, and cannot be seen as a panacea for access inequities. The widespread adaptations to services, while modernising working practices, presented logistical challenges, particularly for smaller organisations, as well as personal challenges for staff and volunteers. However, efficiency and cost effectiveness were reported to increase, and there were valuable lessons learnt for future provision.

On the basis of the study findings, we recommend that:

- Online support provision is carefully designed for different client groups and face-to-face support continues to be provided alongside remote options, recognising that remote methods do not work for everyone and support needs to be tailored to the individual.
- Governments, bereavement organisations and academics create and implement evidence-based ‘digital health toolkits’ to facilitate the provision of effective, appropriate, safe online bereavement support, as have been developed in other fields.^65,66^
- Health systems reduce digital exclusion by supporting the most disadvantaged members of the community to access technology and training to enable them to benefit from remote bereavement support, helping to meet policy guidelines.^29,67^
- Digital technology, and training for bereavement providers to use it, is included in funding arrangements with local authorities and health boards to support the sector.
- Bereavement services provide training on providing remote support, including the psychological impact of home working, and prioritise opportunities for team members to connect and sustain each other.

As leaders of the New York State public mental health system stated in December 2020: ‘when something precious that is complex and fragile suffers a blow that threatens its very existence, do not waste time trying to put it back together as it was. Be creative and innovative to build something stronger and more resilient.’^68^ Our study findings demonstrate the creativity and innovation of bereavement service providers; we must heed these recommendations to ensure that the legacy of the pandemic is a stronger, more resilient and equitable bereavement sector.

## Supplemental Material

sj-docx-1-pmj-10.1177_02692163251383324 – Supplemental material for Shifting to online and telephone bereavement support provision during the COVID-19 pandemic: A mixed methods study of bereavement service provider perspectives and lessons learnt for current practice Supplemental material, sj-docx-1-pmj-10.1177_02692163251383324 for Shifting to online and telephone bereavement support provision during the COVID-19 pandemic: A mixed methods study of bereavement service provider perspectives and lessons learnt for current practice by Lucy E. Selman, Jenny Birchall, Eileen J. Sutton, Tracey Stone, Renata Medeiros Mirra, Emma Gilbert, Mirella Longo, Kathy Seddon, Anne M. Finucane, Alison Penny, Anthony Byrne and Emily Harrop in Palliative Medicine

sj-docx-2-pmj-10.1177_02692163251383324 – Supplemental material for Shifting to online and telephone bereavement support provision during the COVID-19 pandemic: A mixed methods study of bereavement service provider perspectives and lessons learnt for current practice Supplemental material, sj-docx-2-pmj-10.1177_02692163251383324 for Shifting to online and telephone bereavement support provision during the COVID-19 pandemic: A mixed methods study of bereavement service provider perspectives and lessons learnt for current practice by Lucy E. Selman, Jenny Birchall, Eileen J. Sutton, Tracey Stone, Renata Medeiros Mirra, Emma Gilbert, Mirella Longo, Kathy Seddon, Anne M. Finucane, Alison Penny, Anthony Byrne and Emily Harrop in Palliative Medicine
